# Current practice and awareness of perioperative do-not-attempt-resuscitation orders: a single-center retrospective survey and complete questionnaire survey

**DOI:** 10.1007/s00540-024-03447-w

**Published:** 2024-12-25

**Authors:** Keisuke Shimizu, Kyoko Komatsu, Hiroshi Uchida, Mizuki Nawata, Ryo Kubota

**Affiliations:** Department of Anesthesiology, Tokyo Metropolitan Institute for Geriatrics and Gerontology, 35-2, Sakae-Cho, Itabashi-Ku, Tokyo 173-0015 Japan

**Keywords:** Do-not-attempt-resuscitation order, Advance care planning, General anesthesia, Automatic suspension

## Abstract

**Purpose:**

We investigated whether patients who have been issued a do-not-attempt-resuscitation order (DNAR) preoperatively (hereafter, DNAR patients) are informed of the DNAR code change when they undergo anesthesia. We also conducted a survey of the awareness of medical staff regarding perioperative DNARs, and investigated the current situation at a single-center in Japan.

**Methods:**

For DNAR patients managed by anesthesiologists from January 2019 to September 2022, we retrospectively investigated whether the patient was informed of the DNAR code change or the DNAR was automatically suspended without explanation. Next, in July 2023, a questionnaire survey on perioperative DNARs was conducted among all medical staff at our center.

**Results:**

Among the 4,164 cases managed by anesthesiologists during the study period, 100 DNAR patients (2.4%) were identified. Of these, 27 patients received an explanation about the DNAR code change before surgery. Multivariate analysis showed that female patients (odds ratio [OR] 5.3, 95% confidence interval [CI] 3.8–6.7; *p* = 0.023) and patients with low Barthel Index (OR 0.98, 95% CI 0.96–0.99; *p* = 0.010) tended to receive explanations about DNAR code changes. In the questionnaire survey, 25% of the 1,051 respondents answered that DNAR code changes should be explained to patients before surgery.

**Conclusion:**

In clinical practice, 27% of DNAR patients were informed of DNARs code change before surgery. Perioperative advance care planning should be further promoted in clinical practice by creating guidelines and training programs regarding perioperative DNARs.

**Supplementary Information:**

The online version contains supplementary material available at 10.1007/s00540-024-03447-w.

## Introduction

Elderly patients often have many comorbidities and the prognosis of their underlying disease is often limited. During hospitalization or outpatient care, a do-not-attempt-resuscitation order (DNAR) is often adopted for the event of cardiac arrest, meaning that the decision is made to allow the patient to die following the natural course without performing cardiopulmonary resuscitation (CPR). When a patient with a DNAR (hereafter, DNAR patient) undergoes surgery under anesthesia in the operating room, the issue of how to handle preoperative DNARs is difficult.

The American Society of Anesthesiologists (ASA) has established ethical guidelines on how to behave when administering anesthesia to DNAR patients [1]. According to these guidelines, the fact that a preoperative DNAR is automatically suspended when DNAR patients undergo anesthesia is undesirable from the perspective of the right of the patient to self-determination. The guidelines state that surgeons and anesthesiologists should discuss the DNAR with the patient and their family preoperatively and, in principle, change the DNAR code during the operation. In a related study, a systematic review of this topic published in 2021 widely discussed how anesthesiologists and surgeons deal with DNAR patients in the perioperative period and their level of awareness regarding DNAR protocols [2].

In Japan, no guidelines currently address how to handle DNARs during the perioperative period, and research on the subject is lacking. One factor contributing to this situation is likely the relatively low awareness among patients of the concept that patients should have a say in their own treatment plan compared to other countries, and historically it has been difficult to develop a medical culture regarding advance care planning (ACP) including perioperative DNARs. In some facilities, resuscitation orders such as DNARs should be suspended unconditionally in the event of surgery or general anesthesia [3]. In such cases, the thinking is that attempts at resuscitation will be performed in the operating room to the full extent possible. The principle of automatically suspending DNAR during surgery exists because cardiac arrests occurring during surgery are promptly reversible, well monitored, and readily accessible to sufficient response in the operating room. Good outcomes can, therefore, be expected from the application of resuscitation measures [4].

In 2017, the Ministry of Health, Labour, and Welfare created guidelines regarding ACP called “Life Conferences,” which encourage elderly individuals to actively think about the medical care they would like to receive if they become ill in the future. A study in Japan found that the percentage of DNAR patients before a rapid response system activation increased from 8.0% in 2014 to 12.4% in 2020 [5]. Since Japan has become an increasingly aging society, guidelines now urgently need to be developed regarding perioperative DNARs as well. The choice to place a DNAR is undoubtedly an aspect of patient self-determination that merits respect, and changing the DNAR code without providing a detailed explanation to the patient does not seem consistent with an ethically standard of medical care. This study first examined electronic medical records to determine whether patients with a DNAR issued at our facility who underwent surgery managed by an anesthesiologist received an explanation about the initial DNAR needing to be reconsidered perioperatively. Second, based on those results, we investigated factors related to DNAR reconsideration using a questionnaire survey of all medical staff at our hospital on attitudes toward end-of-life care and the handling of perioperative DNARs. The survey included not only doctors and nurses, but also other medical staff, and examined general trends by the type of profession.

## Materials and methods

Our hospital specializes in acute care for elderly patients, and the average age of patients undergoing surgery is over 80 years old. Regarding end-of-life advance directives, there are four types of documents: “documents regarding life-sustaining treatment” and documents to change them, and “documents regarding cardiopulmonary resuscitation” and documents to change them. If a patient has an advance directive in place, the details are entered in a specified format in the electronic medical record. The system for handling DNARs is, thus, thought to be more well-established compared to other general hospitals, and the clinical practice when a DNAR patient undergoes surgery is thought to be more easily visualized. The present study comprised two parts. The first was a retrospective survey using the electronic medical record system at our hospital. The second part was a questionnaire administered to all medical staff at our hospital.

### Retrospective survey

The first study period was from January 2019 to September 2022. During that period, patients were included if they had a DNAR for their original disease issued at our hospital and then underwent surgery for worsening of the original disease or for a different disease under general or regional anesthesia under the management of anesthesiologists. Cases in which an anesthesiologist was not involved (such as local anesthesia administered by the surgeon) were not included in this study.

The following data were collected from the electronic medical record system at our hospital. Main outcomes were about the original preoperative DNAR and the explanation of the DNAR change. First, regarding the original preoperative DNAR, we determined the date on which the DNAR was obtained, who explained the initial DNAR, whether the patient participated in the discussion when the initial DNAR was placed (if the patient was unconscious, the patient was considered to have not participated), and the contents of the DNAR (the original disease of DNAR patients and the patient’s preference as to resuscitation measures). We then confirmed the description of the explanations given by the surgeon or anesthesiologist regarding the preoperative DNAR, or whether no explanation was specifically given. Regarding other patient background characteristics, we collected information on age, sex, body weight, ASA physical status (ASA-PS), Barthel Index on admission and at discharge [6], emergency or elective surgery, type of anesthesia, operation record, postoperative prognosis, and postoperative code status (DNAR or not). In our hospital, regardless of the method of anesthesia, when performing surgery in the operating room, DNAR codes ordered before surgery are suspended according to the internal rules of the hospital. Therefore, if any explanations of the DNAR have not been implemented by the anesthesiology department or other department, this survey treated the DNAR as automatically suspended.

All DNAR patients were divided into the group in which an explanation was given before the DNAR was changed preoperatively (Change group) and the group in which the DNAR was automatically suspended (Auto group). For statistical analyses, we used Student’s *t* test or the Mann–Whitney U-test to examine the significance of differences for continuous variables and the Chi-square test or Fisher’s exact test for categorical variables. To assess associations between the explanation of DNAR change and patient factors, univariate and multivariate logistic regression analyses were conducted. For multivariate logistic regression analysis, we included the following covariates in the model: age, body weight, sex, and preoperative Barthel Index, as factors associated with DNAR acquisition [4]. The results of analyses are presented as adjusted odds ratios (ORs) with 95% confidence intervals (CIs). Values of *p* < 0.05 were considered statistically significant. SPSS software (IBM, Chicago, Illinois) was used for all statistical analyses. If values were missing, those items were excluded from the statistical analysis. Information was obtained from electronic medical records, so no human material was involved. Although this represents sensitive individual information, since a considerable amount of time had passed since admission and many procedures are in place to obtain consent, information disclosure documents and an opt-out format were adopted. The study related to the retrospective survey was approved by the ethics committee of our institution (approval no. R22-016; date of approval, June 2022).

### Questionnaire survey

In July 2023, a questionnaire was administered to all medical staff regarding the topics of DNARs and ACP. The deadline for returning the questionnaire was 1 month, and a reminder was sent to all staff by e-mail 5 days in advance by the implementing department. Respondents were first trained using e-learning materials, and at the end of the training, the contents of the questionnaire items were written on paper and submitted.

The questionnaire was provided to doctors and nurses, and available for completion by other staff members. We broadly categorized survey respondents into surgeons, internists, other doctors, nurses, other medical staff and office workers. Affiliated departments for surgeons included gastroenterological surgery, orthopedic surgery, cardiovascular surgery, urology, neurosurgery, thoracic surgery, otolaryngology, and oral and maxillofacial surgery. Affiliated departments for internists included general geriatrics, gastroenterology, cardiology, respiratory medicine, diabetes, neurology, collagen disease and rheumatology, nephrology, hematology, palliative care, and infectious diseases. Affiliated departments for “other doctors” included anesthesiology, ophthalmology, dermatology, psychiatry, diagnostic radiology, radiotherapy, pathology, clinical laboratory department, and research institutes. For nurses, we did not differentiate between ward nurses and outpatient nurses, whereas nursing assistants were classified as other medical staff. Other medical staff also included pharmacists, psychologists, clinical laboratory technicians, hygienists, orthoptists, nutritionists, physiotherapists, clinical engineers, radiologists, and paramedics. Office workers included general office staff, medical office staff, clerks, social workers, and medical manufacturer employees.

The full questionnaire survey is shown in Online Resource 1. Some questionnaire items were based on the contents of an awareness survey administered to nurses conducted mainly by the Japanese Society of Intensive Care Medicine regarding instructions for not attempting resuscitation [7]. The questionnaire items regarding perioperative DNARs are Item 4 of Question 6 below.

Question 6: For each of the following actions or thoughts regarding a DNAR, please answer yes if you agree or no if you disagree.

1. I have considered a DNAR for an elderly patient who was in poor condition and brought to the emergency room.
2. A DNAR obtained during the previous hospitalization should be invalid during the current hospitalization.
3. Although a patient had a disease that was suitable for surgery, if the patient had a DNAR code, I decided on conservative treatment rather than surgery for the patient.
4. Even for patients with a DNAR, if surgery is to be performed under general anesthesia, the DNAR code should be changed.

These items of Question 6 were created based on issues related to clinical judgment regarding DNARs, that are often raised on discussion in the medical safety in our hospital. In this study, we specifically examined the item in the questionnaire regarding perioperative DNARs, which was “Even for patients with a DNAR, if surgery is to be performed under general anesthesia, the DNAR code should be changed” (Question 6, Item 4 above). This questionnaire survey was conducted as part of the comprehensive training for staff at our hospital. We, therefore, did not obtain individual consent forms from staff. The study related to the questionnaire survey was approved by the research ethics committee of our institution (approval no. R22-016; date of approval, October 2023).

## Results

### Retrospective survey

**Fig. 1:**
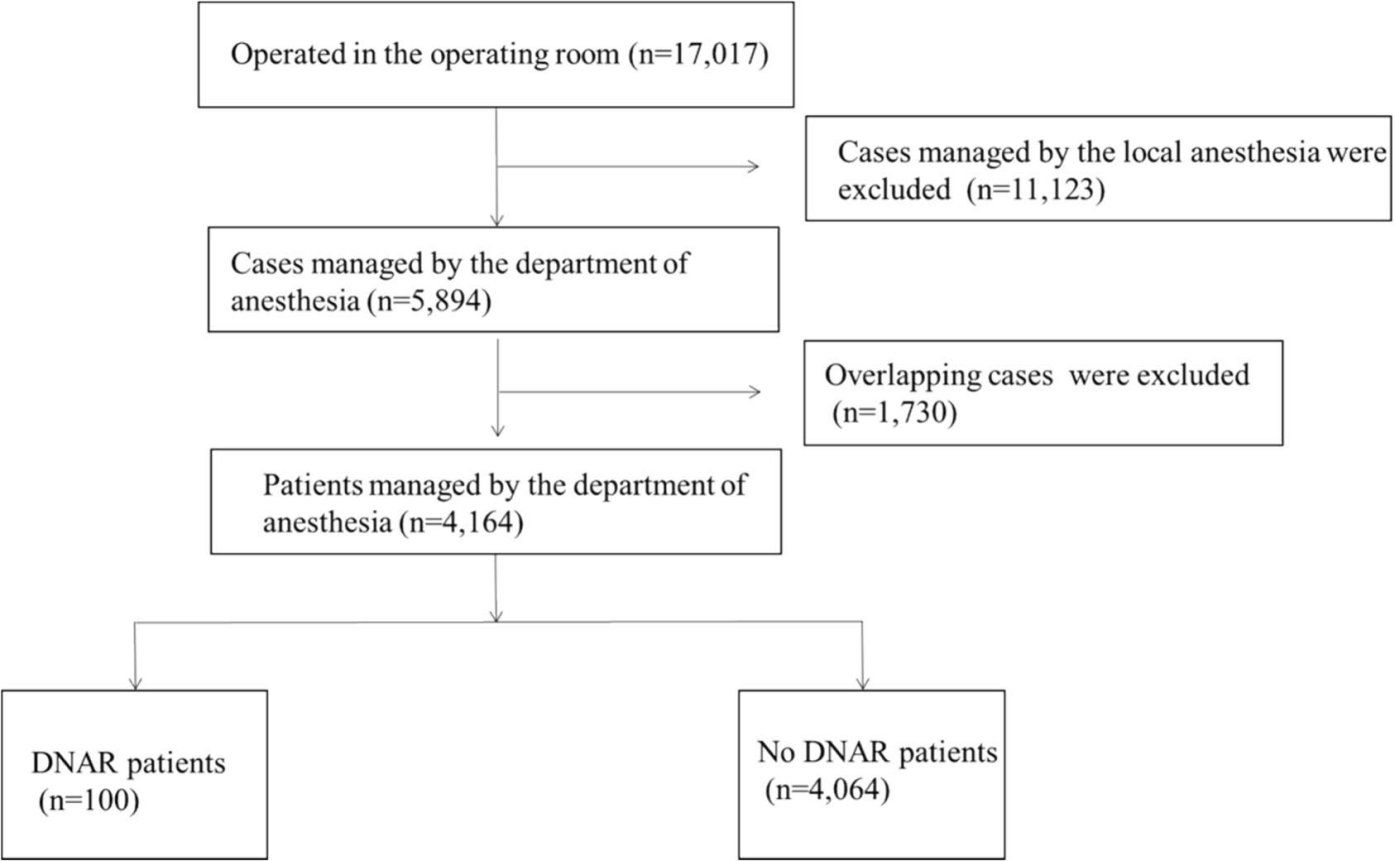
Flow chart for patient inclusion in this retrospective survey. “DNAR patients” means patients issued a DNAR order before surgery

Between January 2019 and September 2022, a total of 17,017 cases were operated on in our operating room. Of these, a total of 5,894 cases were managed by the Department of Anesthesiology under general or regional anesthesia. After excluding overlapping cases in which surgery had been performed several times, 4,164 patients were identified, of whom 100 cases (2.4%) had a DNAR in place before the date of surgery (Fig. 1).

Background characteristics of DNAR patients are shown in Table 1. Mean patient age was 82.4 years (standard deviation [SD]: 9.7 years), 53 patients (53%) were male, mean weight was 51.3 kg (SD: 11.3 kg), and ASA-PS was 2, 3, or ≥ 4 in 49, 47, and 4 cases, respectively. Mean preoperative Barthel Index was 38.9 (SD: 36.1). Regarding the operation, surgery was elective in 80 cases and emergency in 20 cases. Mean operation time was 107.6 min (SD: 99.5 min), and anesthesia was general in 66 cases and regional in 34 cases. Looking at the 100 cases with a DNAR in place preoperatively, the surgery was gastrointestinal in 24%, urological in 19%, cardiovascular in 19%, and orthopedic in 16%.

**Table 1:** Background characteristics and details of preoperative DNAR and postoperative prognosis for DNAR patients

Patient background characteristics	*n* = 100
Age (years)	82.4 (SD 9.7)
Sex (male/female)	53/47
Body weight (kg)	51.3 (SD 11.3)
ASA-PS (2/3/ ≥ 4)	49/47/4
Preoperative Barthel Index	38.9 (SD 36.1)
Elective/emergency	80/20
Operation time (min)	107.6 (SD 99.5)
General/regional anesthesia	66/34
Preoperative DNAR	
Doctor explaining DNAR	
Internist	50
Emergency doctor	25
Surgeon	23
Other	2
Participation in person (yes/no)	46/54
Oxygenation preference	97%
Vasopressor preference	63%
Prognosis	
Cardiopulmonary arrests during operation	0
Length of hospital stay (days)	22.6 (SD 18.0)
Postoperative mortality rate	
1 month	8%
6 months	12%

“Participation in person” means that the patient took part in the explanation of the initial DNAR. “Oxygenation preference” means that the patient consented to oxygen administration should the need arise. “Vasopressor preference” means that the patient consented to administration of a vasopressor should the need arise

*ASA-PS* American Society of Anesthesiologists-Physical Status, *DNAR* do-not-attempt-resuscitation order, *SD* standard deviation

The initial DNAR was placed by an internist in 50 cases, emergency physician in 25 cases, surgeon in 23 cases, and others in 2 cases. Of the remaining 2 cases, one had a living will from the Japan Society for Dying with Dignity, and the other had a DNAR obtained at another hospital (details unknown). In 54 cases, the patient did not participate in the explanation given at the time the initial DNAR was obtained. The type of disease that caused the DNAR was cancer in 10 cases, respiratory disease in 28 cases, cerebrovascular disease in 28 cases, cardiovascular disease in 11 cases, renal disease in 12 cases, and other reasons in 11 cases. Other reasons included advanced age, malnutrition and severe anemia. Four patients received a DNAR due to COVID-19 infection. In 38 cases, the surgery was due to the same disease that resulted in the DNAR being placed.

In 27 cases (27%), the initial DNAR was changed through preoperative explanation. Of these, 11 cases were changed by an anesthesiologist, and 19 cases by doctors in other departments (3 cases were changed by both an anesthesiologist and a doctor in other departments). Of the 19 cases discussed for DNARs by doctors in other departments, 11 cases were explained by the surgeons performing the operation and 8 cases by the attending physician managing the original disease. In the remaining 73 cases (73%), no specific explanation was given as to the DNAR, and the DNAR was considered automatically suspended.

Of the 100 DNAR patients, none experienced cardiac arrest during surgery. Mean hospital stay after the operation was 22.6 days (SD: 18.0 days), and 1- and 6-month survival rates were 8% and 12%, respectively. Postoperative code was identified in 36 patients based on electronic medical records. All of the codes were DNAR, and for the remaining 64 cases, there was no specific explanation and the postoperative code was unknown. The median period during which DNAR was suspended was 170 days (interquartile range: 332 days).

Next, we examined differences in background factors between the Change group (27 cases) and Auto group (73 cases). Between Change group and Auto group, the proportion of women (70% vs. 38%, *p* = 0.004) and preoperative Barthel Index (35.4 vs. 54.5, *p* = 0.024) differed significantly. No significant differences were seen in length of hospital stay after surgery or 1- or 6-month mortality rates between the Change and Auto groups (Table 2). To further examine background factors contributing to explanations for changing the initial DNAR, univariate logistic regression analysis identified female sex (OR 3.8, 95% CI 2.9–4.8; *p* = 0.006) and low preoperative Barthel Index (OR 0.98, 95% CI 0.96–0.99; *p* = 0.007) as factors associated with changing the code. Adjusting for age and body weight, multivariate logistic regression analysis also revealed female sex (OR 5.3, 95% CI 3.8–6.7; *p* = 0.023) and low Barthel Index (OR 0.98, 95% CI 0.96–0.99; *p* = 0.010) as significant factors (Table 3).

**Table 2:** Comparison of background characteristics and outcomes between the change group and Auto group

	Change group (*n* = 27)	Auto group (*n* = 73)	*p* value
Background characteristics			
Sex			
Male/female	30%/70%	62%/38%	0.004*
Age (years old)	85.2	81.3	0.07
Body weight (kg)	48.1	52.5	0.09
Preoperative Barthel Index	35.4	54.5	0.024*
Participation in person	44.4%	46.6%	0.85
Emergency operation	18.5%	20.5%	0.82
Outcomes			
Length of hospital stay (day)	25.3	21.5	0.35
Postoperative 1 month mortality	11.1%	6.8%	0.44
Postoperative 6-month mortality	14.8%	11.0%	0.73

“Change group” is the group for whom an explanation was given and the initial DNAR was changed preoperatively, and “Auto group” is the group for whom the DNAR was automatically suspended without explanation. “Participation in person” means that the patient took part in the explanation of the initial DNAR. *p < 0.05

**Table 3:** Univariate logistic regression analysis and multivariate logistic regression analysis adjusted for age, body weight, sex and preoperative Barthel Index regarding DNAR code change

	Univariate		Multivariate	
	OR [95%CI]	p value	OR [95%CI]	p value
Age	1.05 [0.99–1.11]	0.07	1.04 [0.98–1.10]	0.22
Body weight	0.96 [0.92–1.01]	0.09	1.03 [0.96–1.10]	0.36
Sex (female)	3.8 [2.9–4.8]	0.006*	5.3 [3.8–6.7]	0.023*
Preoperative Barthel Index	0.98 [0.96–0.99]	0.007*	0.98 [0.96–0.99]	0.010*

We included the following covariates in the model: age, body weight, sex and preoperative Barthel Index, as factors thought to show the background of the patient and considered to be associated with DNAR acquisition. “Univariate” means univariate logistic regression analysis and “Multivariate” means multivariate logistic regression analysis. *OR* odds ratio, *95% CI* 95% confidence interval. *p < 0.05

### Questionnaire survey

**Fig. 2:**
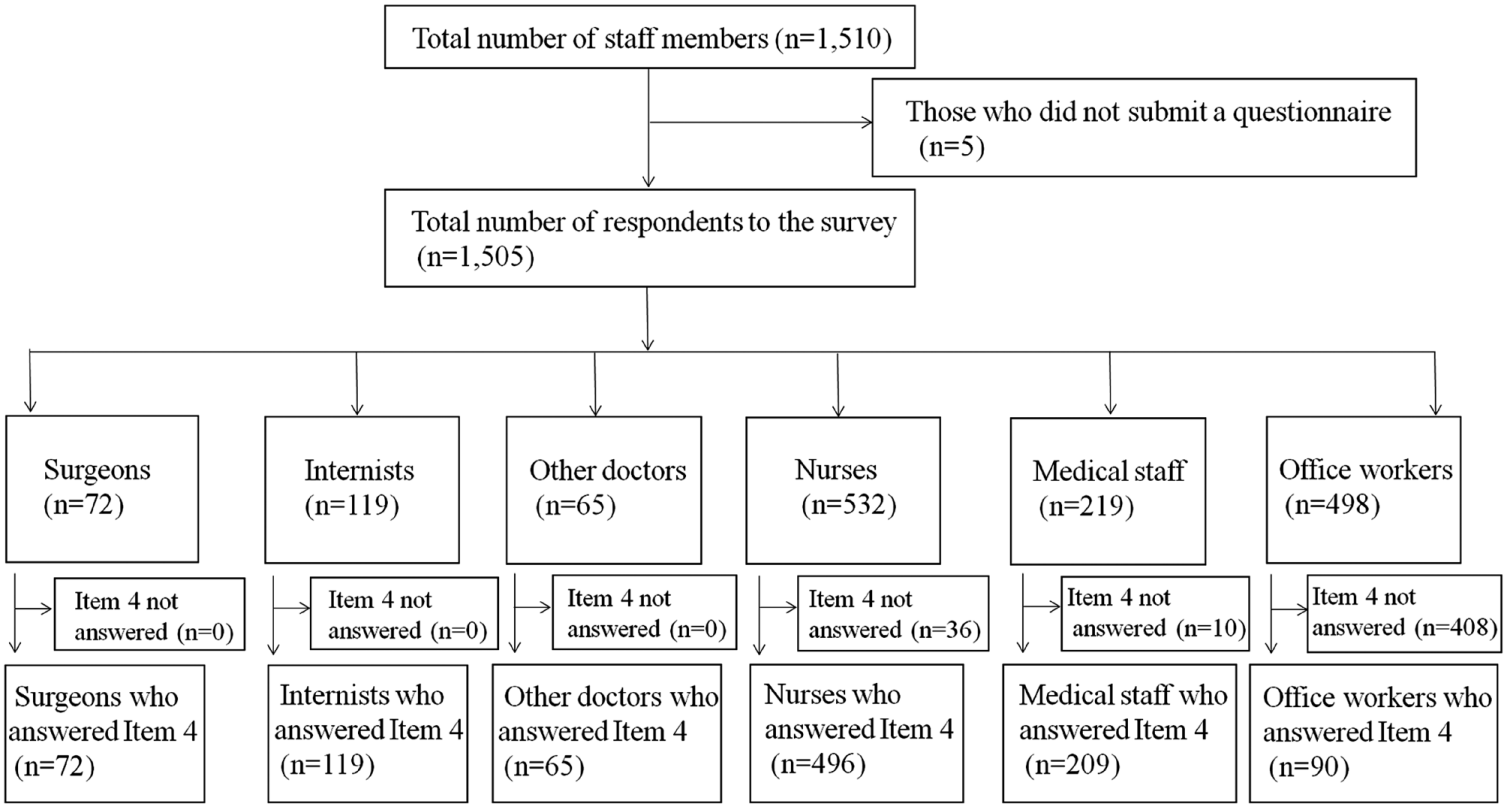
Flow chart for participant inclusion in the questionnaire survey

In total, 1,505 respondents returned to a survey, of whom 1051 (69.8%) responded to Item 4. These 1,051 respondents comprised 72 surgeons, 119 internists, 65 other doctors (including anesthesiologists), 496 nurses, 209 other medical staff, and 90 office workers (Fig. 2).

Overall, 25% of respondents answered that DNARs should be changed after explanations have been given to DNAR patients before the operation. By profession, these percentages were 39% among surgeons, 29% among internists, 35% among other doctors, 27% among nurses, 15% among other medical staff, and 11% among office workers. Regarding anesthesiologists, 9 of the 15 responded that the preoperative DNAR should be reviewed. As to the full questionnaire survey shown in Online Resource 1, the percentage of responses to all questions by profession is shown in Table 4 in Online Resource 2 and answers to each questionnaire items by the type of profession are summarized in Table 5 in Online Resource 2.

## Discussion

This study investigated how many patients had a DNAR in place preoperatively and how many of them received an explanation about changing the DNAR. At the same time, we conducted a survey of hospital staff regarding their awareness of perioperative DNARs. Previous studies in other countries have shown that 1.4–13.6% of patients had preoperative DNARs [4, 8–10], 18–60% had DNARs lifted before surgery [11, 12], and 13–30% died by 1 month after surgery [13, 14]. According to our data, DNAR patients accounted for 2.4% of all patients managed by anesthesiologists, and 27% of these patients had DNARs changed after receiving an explanation. Compared to previous studies, the percentage of DNAR patients was relatively low, but this may be because patients who actually had intentions for a DNAR did not have this information clearly recorded in the medical chart, or because this information was difficult to extract even if recorded. In addition, while laws exist regarding advance directives and DNARs, such as the Patient Self-Determination Act (enacted in 1990) in the United States, no equivalent law exists in Japan, which may be one reason for the low rate of DNARs among patients in general [15]. Some research has shown that the percentage of people with written advance directives was 36.7% in the United States, but only 2.6% in Japan [16, 17].

However, the percentage of cases in which DNARs were changed with explanation was only 27%, comparable to rates in other countries. The first reason is that, particularly in emergency cases, limited time is available to talk with patients and their families, so little room exists to ascertain the value of the patient. The second reason is that anesthesiologists are accustomed to the routine work of explaining anesthesia methods to patients, but have little experience in ACP involving other professions [4]. Factors such as “low ADL” and “female sex” were extracted as reasons for the high rate of DNAR suspension by preoperative explanations. As far as we have examined, no similar research has been conducted on DNAR suspension, so we compared these results with results from previous research on DNAR acquisition. Regarding ADL, a study on patients hospitalized in geriatric wards showed that DNAR patients had a significantly lower Barthel Index than those without a DNAR [18]. Many patients with poor preoperative ADL are considered to be in poor overall condition, and such patients are likely to experience a sudden change during surgery, so medical professionals are speculated to be more likely to be aware of the need to confirm the policy in advance in case of such an event. Regarding “female sex,” more female patients than male patients reportedly have DNARs in the first place [19]. The reasons for this may involve the longer lifespan of women, making medical decisions such as DNARs more likely to be made by surrogate decision makers in the case of women, or that women are better than men at conveying their wishes to their families. A Japanese study also showed that social interaction decreases in elderly male patients over 80 years old compared to females [20], so DNARs may be reviewed in female patients when explanations about surgery are given to family and friends. However, the definitive reasons remain unclear.

In the questionnaire survey of medical staff, 25% of respondents said that DNAR patients should be asked to confirm their wishes regarding treatment in the event of a sudden change in condition during the perioperative period. The reason for this result might be that, since DNARs are generally suspended during the perioperative period, some medical professionals did not consider that there might be a need to mention this to patients and their family. A similar questionnaire survey was conducted in the United States in 1990 and showed that 40% of anesthesiologists explained the need to reconsider preoperative DNARs [21]. However, a 2013 survey conducted in the United States showed that 82% of anesthesiologists, 66% of internists, and 62% of surgeons explained and reconsidered DNAR code changes [22]. The ASA ethical guidelines on DNARs were created in 2001, and the guidelines may have played a role in promoting reconsideration of perioperative DNARs.

One limitation of this study was that DNAR patients who were considered for surgery but who did not undergo surgery were not included. This likely included cases in which patients did not wish to receive surgery after listening to the explanation from the anesthesiologist. For such patients, an important role of the anesthesiologist is to perform a preoperative evaluation to determine whether the patient is capable of enduring surgery and to explain the situation to the patient. These patients should have been included in the study, but due to the method of data collection, obtaining data on patients who did not undergo surgery was impossible. Another limitation of the questionnaire survey was that although the submission rate was almost 100% because the questionnaire itself was conducted as part of a training program in our hospital, items related to perioperative DNAR were optional, particularly for non-healthcare professionals. Therefore, some selection bias may have been present. For example, nurses who did not respond to Item 4 in Question 6 may have thought that they were unable to make a decision because the patient’s wishes were unclear just by reading the question, and if that is the case, the percentage of nurses who thought that a preoperative DNAR should be changed may be even higher. Finally, this study was a single-center retrospective study in a hospital specializing in elderly patients. Even if the number of elderly patients increases in hospitals throughout Japan in the future, we cannot consider the results of this study directly applicable to small hospitals or clinics in rural areas, and the external validity is clearly limited.

This study retrospectively examined how DNARs are actually handled when administering anesthesia to DNAR patients in our hospital, and surveyed medical professionals to investigate factors related to DNAR reconsideration. In Japan, awareness of the need for preoperative explanation for DNAR patients in the perioperative period is not currently widespread. To respect the wishes of the patient, creating guidelines originating in Japan and conducting step-by-step awareness-raising activities regarding ACP may be meaningful.

## Supplementary Information

Below is the link to the electronic supplementary material.

Supplementary file1 (DOCX 17 KB)

Supplementary file2 (DOCX 22 KB)

## Data Availability

The datasets related to this study are available from the corresponding author on reasonable request.

## References

[1] Statement on Ethical Guidelines for the Anesthesia Care of Patients with Do-Not-Resuscitate Orders. American Society of Anesthesiologists Committee on Ethics. 2023 Oct (original approval: October 17, 2001). https://www.asahq.org/standards-and-practice-parameters/statement-on-ethical-guidelines-for-the-anesthesia-care-of-patients-with-do-not-resuscitate-orders. Accessed 8 Aug 2024.

[2] Cushman T, Waisel DB, Treggiari MM. The role of anesthesiologists in perioperative limitation of potentially life-sustaining medical treatments: a narrative review and perspective. Anesth Analg. 2021;133(3):663–75. 34014183 10.1213/ANE.0000000000005559

[3] Minooka M. The fate of resuscitation orders- ethics of DNAR for medical professionals (main text in Japanese). World Planning. 2012. p. 52.

[4] Nolan JP, Soar J, Kane AD, Moppett IK, Armstrong RA, Kursumovic E, Cook TM. Peri-operative decisions about cardiopulmonary resuscitation among adults as reported to the 7th National Audit Project of the Royal College of Anaesthetists. Anaesthesia. 2024;79(2):186–92. 37991058 10.1111/anae.16179

[5] Tsuji T, Sento Y, Sobue K. Trends in DNAR orders for deteriorating patients in Japan. J Anesth. 2024;38:288–90. 38135844 10.1007/s00540-023-03298-x

[6] Wade DT, Collin C. The Barthel ADL Index: a standard measure of physical disability? Int Disabil Stud. 1988;10(2):64–7. 3042746 10.3109/09638288809164105

[7] Clinical Ethics Committee, Japanese Society of Intensive Care Medicine. Nationwide assessment of Japanese ICU nurse's understanding of Do Not Resuscitate (DNR) Order and use of Japanese Society Intensive Care Medicine (JSICM) Guideline. J Jpn Soc Intensive Care Med. 2020;27(3):231–43.

[8] Brovman EY, Pisansky AJ, Beverly A, Bader AM, Urman RD. Do-not-resuscitate status as an independent risk factor for patients undergoing surgery for hip fracture. World J Orthop. 2017;8:902–12. 29312849 10.5312/wjo.v8.i12.902 PMC5745433

[9] Siracuse JJ, Jones DW, Meltzer EC, Graham AR, Salzler GG, Connolly PH, Schneider DB, Meltzer AJ. Impact of “do not resuscitate” status on the outcome of major vascular surgical procedures. Arch Surg. 2015;29(7):1339–45. 10.1016/j.avsg.2015.05.014 26169461

[10] Scarborough JE, Pappas TN, Bennett KM, Lagoo-Deenadayalan JE. Failure-to-pursue rescue: explaining excess mortality in elderly emergency general surgical patients with preexisting “do-not-resuscitate” orders. Ann Surg. 2012;256(3):453–61. 22868360 10.1097/SLA.0b013e31826578fb

[11] Wenger NS, Greengold NL, Oye RK, Kussin P, Phillips RS, Desbiens NA, Liu H, Hiatt JP, Teno JM, Connors AF. Patients with DNR orders in the operating room: surgery, resuscitation, and outcomes. SUPPORT Investigators. Study to understand prognoses and preferences for outcomes and risks of treatments. J Clin Ethics. 1997;8(3):250–7. 9436083

[12] McBrien ME, Kavanagh A, Heyburn G, Elliott JR. “Do not attempt resuscitation” (DNAR) decisions in patients with femoral fractures: modification, clinical management and outcome. Age Ageing. 2013;42(2):246–9. 22832379 10.1093/ageing/afs096

[13] Brovman EY, Walsh EC, Burton BN, Kuo CE, Lindvall C, Gabriel RA, Urman RD. Postoperative outcomes in patients with a do-not-resuscitate (DNR) order undergoing elective procedures. J Clin Anesth. 2018;48:81–8. 29783184 10.1016/j.jclinane.2018.05.007

[14] Speicher PJ, Lagoo-Deenadayalan SA, Galanos AN, Pappas TN, Scarborough JE. Expectations and outcomes in geriatric patients with do-not-resuscitate orders undergoing emergency surgical management of bowel obstruction. JAMA Surg. 2013;148(1):23–8. 23324836 10.1001/jamasurg.2013.677

[15] Ishinomaki S, Yamanaka I. Indications of DNAR order for CPA cases (in Japanese). Kyukyuigaku. 1999;23:1873–7.

[16] Yadav KN, Gabler NB, Cooney E, Kent S, Kim J, Herbst N, Mante A, Halpern SD, Courtright KR. Approximately one in three US adults completes any type of advance directive for end-of-life care. Health Aff. 2017;36(7):1244–51. 10.1377/hlthaff.2017.0175 28679811

[17] Inoue M, Hanari K, Hamano J, Gallagher J, Tamiya N. Current engagement in advance care planning in Japan and its associated factors. Gerontol Geriatr Med. 2019;5:2333721419892694. 31903410 10.1177/2333721419892694 PMC6926984

[18] Chien YW, Chun HJ, Yun SC, Tzu JF, Yu HW, Ming TW. Factors associated with do-not-resuscitate document completion among patients hospitalized in geriatric ward. BMC Geriatr. 2021;21:472. 34433419 10.1186/s12877-021-02407-3 PMC8386141

[19] Sarah MP, Bonnie JS, Adit AG, Anne VG, Benjamin SA, Stacie LD, Edward PH. Sex differences in “do not attempt resuscitation” orders after out-of-hospital cardiac arrest and the relationship to critical hospital interventions. Clin Ther. 2019;41(6):1029–37. 31047712 10.1016/j.clinthera.2019.03.017 PMC7213038

[20] Kawai H, Ejiri M, Ito K, Fujiwara Y, Ihara K, Hirano H, Sasai H, Kim H, Obuchi S. Social interaction trajectories and all-cause mortality in older adults: the Otassha study. Front Public Health. 2023;11:1248462. 37674679 10.3389/fpubh.2023.1248462 PMC10477580

[21] Clemency MV, Thompson NJ. “Do not resuscitate”(DNR) orders and the anesthesiologist: a survey. Anesth Analg. 1993;76:394–401. 8424522

[22] Burkle CM, Swetz KM, Armstrong MH, Keegan MT. Patient and doctor attitudes and beliefs concerning perioperative do not resuscitate orders: anesthesiologists’ growing compliance with patient autonomy and self determination guidelines. BMC Anesthesiol. 2013;13:2. 23320623 10.1186/1471-2253-13-2 PMC3548687

