# Supplementary material for: Current practice and awareness of perioperative do-not-attempt-resuscitation orders: a single-center retrospective survey and complete questionnaire survey
Source: J Anesth. 2024 Dec 25;39(2):223–30. doi: 10.1007/s00540-024-03447-w (PMC12494614; doi:10.1007/s00540-024-03447-w)
Supplement: Supplementary file 1 — Supplementary file1 (DOCX 17 KB) [file 540_2024_3447_MOESM1_ESM.docx]

**Online Resource 1**

**Title:**

Current practice and awareness of perioperative do-not-attempt-resuscitation orders –a single-center retrospective survey and complete questionnaire survey–

**Journal name:**

Journal of Anesthesia

**Author names:**

Keisuke Shimizu^1^, Kyoko Komatsu^1^, Hiroshi Uchida^1^, Mizuki Nawata^1^, Ryo Kubota^1^

**Affiliation:**

^1^Department of Anesthesiology, Tokyo Metropolitan Institute for Geriatrics and Gerontology

**Corresponding author:**

Keisuke Shimizu

**Email**: kaigai.shimmy@gmail.com

Full contents of the questionnaire survey

Some elderly patients do not wish to receive aggressive treatment if their condition suddenly changes or their heart stops. Making clear that resuscitation measures such as cardiac massage should not be performed when the heart stops is called a Do-Not-Attempt-Resuscitation order (DNAR).

Question 1 What kind of treatment would you like for your elderly family member if they were to develop a serious illness with little chance of recovery?

1. I would like aggressive treatment, and if their heart stops, I would like resuscitation measures such as cardiac massage.

2. I would like treatment as aggressive as possible, but if their heart stops, I would not like resuscitation measures.

3. I would not like aggressive treatment, and would like treatment focused on symptom relief.

4. I don't know.

Question 2 Please circle the following words that you use or hear in your daily life (multiple answers possible)

1. DNAR

2. Full course, natural course

3. BSC (best supportive care)

4. Withhold

5. Advance directive

6. Living will

7. ACP (advanced care planning)

8. I don't know any of them

Question 3 How much do you know about ACP?

1. I am very familiar with it

2. I think I know about it

3. I am not very familiar with it

4. I don't know anything about it

Please only answer the following questions if you are a medical professional.

Question 4 How often do you come into contact with DNAR patients in your daily work at the hospital?

1. Almost every day

2. A few patients per week

3. A few patients per month

4. Almost never

Question 5 Please circle the items you think should be done for patients with a DNAR policy (multiple options possible)

1. Fluid infusion

2. Admission to the intensive care unit

3. Blood transfusion

4. Administration of vasopressors

5. Endotracheal intubation

6. Cardiac massage

7. ECMO or PCPS

8. None of these

Question 6 For each of the following actions or thoughts regarding a DNAR, please answer yes if you agree or no if you disagree.

1. I have considered a DNAR for an elderly patient who was in poor condition and brought to the emergency room.

2. A DNAR obtained during the previous hospitalization should be invalid during the current hospitalization.

3. Although a patient had a disease that was suitable for surgery, if the patient had a DNAR code, I decided on conservative treatment rather than surgery for the patient.

4. Even for patients with a DNAR, if surgery is to be performed under general anesthesia, the DNAR code should be changed.
