# Supplementary material for: Current practice and awareness of perioperative do-not-attempt-resuscitation orders: a single-center retrospective survey and complete questionnaire survey
Source: J Anesth. 2024 Dec 25;39(2):223–30. doi: 10.1007/s00540-024-03447-w (PMC12494614; doi:10.1007/s00540-024-03447-w)
Supplement: Supplementary file 2 — Supplementary file2 (DOCX 22 KB) [file 540_2024_3447_MOESM2_ESM.docx]

**Online Resource 2**

**Title:**

Current practice and awareness of perioperative do-not-attempt-resuscitation orders –a single-center retrospective survey and complete questionnaire survey–

**Journal name:**

Journal of Anesthesia

**Author names:**

Keisuke Shimizu^1^, Kyoko Komatsu^1^, Hiroshi Uchida^1^, Mizuki Nawata^1^, Ryo Kubota^1^

**Affiliation:**

^1^Department of Anesthesiology, Tokyo Metropolitan Institute for Geriatrics and Gerontology

**Corresponding author:**

Keisuke Shimizu

**Email**: kaigai.shimmy@gmail.com

Table 4 Percentages of responses to all questions in the Online Resource 1 by profession

| Question | 1 | 2 | 3 | 4 | 5 | 6 |
| --- | --- | --- | --- | --- | --- | --- |
| Surgeons | 100% | 100% | 100% | 100% | 100% | 100% |
| Internists | 100% | 100% | 100% | 99.2% | 100% | 100% |
| Other doctors | 100% | 100% | 100% | 98.5% | 100% | 100% |
| Nurses | 100% | 100% | 99.4% | 93.8% | 94.0% | 93.2% |
| Medical staff | 100% | 99.5% | 100% | 95.0% | 95.0% | 95.4% |
| Office workers | 99.8% | 99.8% | 100% | 19.1% | 18.3% | 18.1% |

Table 5 Answers to each question by profession in the Online Resource 1.

| Question | 1 | 2 | 2 | 3 | 4 |  |
| --- | --- | --- | --- | --- | --- | --- |
| Item | 1/2/3/4 | 1/2/3/4 | 5/6/7/8 | 1/2/3/4 | 1/2/3/4 |  |
| Surgeons | 10/18/41/3 | 57/51/53/11 | 40/50/29/3 | 6/25/25/16 | 10/5/17/40 |  |
| Internists | 0/35/80/4 | 118/108/112/28 | 97/104/83/0 | 20/63/29/7 | 66/21/16/15 |  |
| Other doctors | 4/18/40/3 | 57/49/50/13 | 32/46/22/2 | 2/15/33/15 | 11/6/15/32 |  |
| Nurses | 15/105/381/31 | 488/401/306/11 | 369/285/391/25 | 35/315/122/57 | 224/97/102/75 |  |
| Medical staff | 9/65/127/18 | 159/94/79/4 | 84/92/61/31 | 3/48/82/86 | 27/33/41/107 |  |
| Office workers | 51/130/237/79 | 137/63/70/6 | 101/106/59/245 | 2/37/115/344 | 7/8/1/79 |  |
| Question | 5 | 5 | 6 |  |  |  |
| Item | 1/2/3/4 | 5/6/7/8 | 1 | 2 | 3 | 4 |
| Surgeons | 50/9/21/19 | 3/0/0/13 | 37 | 38 | 29 | 28(38.9%) |
| Internists | 107/17/46/47 | 3/1/2/12 | 85 | 50 | 21 | 34(28.6%) |
| Other doctors | 55/17/20/18 | 4/1/1/8 | 37 | 31 | 22 | 23(35.4%) |
| Nurses | 385/41/144/181 | 8/9/6/104 | 291 | 347 | 117 | 136(27.4%) |
| Medical staff | 152/55/71/75 | 17/9/4/41 | 99 | 136 | 85 | 32(15.3%) |
| Office workers | 62/22/15/12 | 7/2/1/18 | 18 | 18 | 52 | 10(11.1) |

Responses to the questions in the questionnaire survey given to all staffs are summarized by profession. For Questions 1, 3 and 4, the number of people who answered each of the items 1 to 4 is indicated. For Questions 2, 5 and 6, the number of respondents who answered "yes" is listed. For Item 4 of Question 6, the percentage is also shown because this is a main result.
